# ReNE: A Cytoscape Plugin for Regulatory Network Enhancement

**DOI:** 10.1371/journal.pone.0115585

**Published:** 2014-12-26

**Authors:** Gianfranco Politano, Alfredo Benso, Alessandro Savino, Stefano Di Carlo

**Affiliations:** 1 Department of Control and Computer Engineering, Politecnico di Torino, Torino, Italy; 2 Consorzio Interuniversitario Nazionale per l′Informatica, Verres (AO), Italy; University of Erlangen-Nuremberg, Germany

## Abstract

One of the biggest challenges in the study of biological regulatory mechanisms is the integration, americanmodeling, and analysis of the complex interactions which take place in biological networks. Despite post transcriptional regulatory elements (i.e., miRNAs) are widely investigated in current research, their usage and visualization in biological networks is very limited. Regulatory networks are commonly limited to gene entities. To integrate networks with post transcriptional regulatory data, researchers are therefore forced to manually resort to specific third party databases. In this context, we introduce ReNE, a Cytoscape 3.x plugin designed to automatically enrich a standard gene-based regulatory network with more detailed transcriptional, post transcriptional, and translational data, resulting in an enhanced network that more precisely models the actual biological regulatory mechanisms. ReNE can automatically import a network layout from the Reactome or KEGG repositories, or work with custom pathways described using a standard OWL/XML data format that the Cytoscape import procedure accepts. Moreover, ReNE allows researchers to merge multiple pathways coming from different sources. The merged network structure is normalized to guarantee a consistent and uniform description of the network nodes and edges and to enrich all integrated data with additional annotations retrieved from genome-wide databases like NCBI, thus producing a pathway fully manageable through the Cytoscape environment. The normalized network is then analyzed to include missing transcription factors, miRNAs, and proteins. The resulting enhanced network is still a fully functional Cytoscape network where each regulatory element (transcription factor, miRNA, gene, protein) and regulatory mechanism (up-regulation/down-regulation) is clearly visually identifiable, thus enabling a better visual understanding of its role and the effect in the network behavior. The enhanced network produced by ReNE is exportable in multiple formats for further analysis via third party applications. ReNE can be freely installed from the Cytoscape App Store (http://apps.cytoscape.org/apps/rene) and the full source code is freely available for download through a SVN repository accessible at http://www.sysbio.polito.it/tools_svn/BioInformatics/Rene/releases/. ReNE enhances a network by only integrating data from public repositories, without any inference or prediction. The reliability of the introduced interactions only depends on the reliability of the source data, which is out of control of ReNe developers.

## Introduction

In the past decade, high-throughput technologies produced a huge amount of biological data. This stimulated the creation of several data repositories attempting to store and integrate biological information and to make it available to researchers. Repositories storing biological pathways and information about microRNA (miRNA) target genes are two well known examples of repositories aiming at representing a set of important regulatory processes that happen at molecular level. Their data are nowadays widely used for computational and experimental research. Scientists in different fields of Bioinformatics and Systems Biology daily refer to these databases to obtain better insights from the high amount of data generated by their experiments, or to perform preliminary hypothesis required to setup new laboratory experiments [1].

miRNAs are short non-coding RNA sequences that regulate gene expression [2]. They are thought to target the 3′ Untranslated Regions (UTRs) of mRNA, disrupting their ability to be translated into proteins and sometimes repressing the expression of the mRNA itself [3]–[6]. miRNA target genes stored in online repositories are usually predicted resorting to pattern matching techniques that pair the seed region of the miRNA (bases 2–8 from the 5′ end of the microRNA) to a cognate mRNA sequence. The nature of the miRNA-mRNA binding is very complex. It is not a perfect match that pairs complementary bases. It is instead a partial match where only few base pairs match. This mechanism makes the discovery of miRNA target genes computationally complex [7], [8]. This generated a large amount of false positive predictions stored in several public repositories, which makes the analysis and the retrieval of useful data more difficult. Among the most addressed repositories for miRNA target genes we can include miRTarBase [9], TargetScan [10], PicTar [11] and miRanda [12]. Moreover, online resources like TargetHUB [13], which integrate data from multiple miRNA repositories, allow users to systematically integrate data from multiple sources.

Biological pathways, also referred to as biological networks, are another very important example of well established biological information stored in public repositories. Biological pathways are the most visual-friendly way to graphically represent the regulatory mechanisms of cellular activities. Compared with the study of individual genes and molecules, biological networks offer a more high-level, informative, and consistent way to deal with and to process complex data. Researchers can analytically inspect relations among entities resorting to the network theory. Moreover, non-trivial patterns, difficult or impossible to be noticed by automatic algorithms, can be sometimes identified by expert researchers through visual inspection of the network [14]. According to Pathguide [15], there are currently 547 online repositories of pathways and molecular interactions. Among them, KEGG [16] and Reactome [17] are two well established databases developed and maintained by dedicated research groups. Other pathway repositories range from community-based repositories such as WikiPathways [18], repositories dedicated to specific cell activities such as the signaling networks contained in the SignaLink2 repository [19] and both free and commercial company-hosted repositories like Ingenuity [20] and BioCarta [21]. Some repositories also try to integrate pathways information from the aforementioned databases in an attempt to provide an uniform way to access those resources (PID [22], Pathway Commons [23]).

With multiple databases available in distinct and sometimes proprietary formats, data integration has become a critical task to allow users to systematically access data in an efficient manner. Integration of pathway data is a critical issue due of the lack of an established and recognized standard for representing the pathway structure, or for exchanging data between different sources. Although pathway description languages such as the Biological Pathways Exchange (BioPAX) format [24] and the Systems Biology Markup Language (SBML) [25] have been proposed as standards for pathway data exchange in XML format, each pathway database is constructed based on its own pathway model. This model basically reflects the designer's view on the selected pathway and is often incompatible with other models. Therefore even simple integrations among different repositories are complex. A coherent and comprehensive way for the analysis and visualization of pathways coming from different databases is still far from being achieved. The integration of miRNA target data in a pathway is instead easier but still important. This is favored by the fact that the atomic unit of information basically corresponds to a miRNA and one of its target genes. Small differences among databases containing information about miRNA target genes are the effect of their proprietary matching/binding scores, and of the third-party databases used for the definition of the single entities (i.e., gene and miRNA id/sequence information).

Pathway visualization is another challenging task in pathway analysis. Online repositories usually provide pathways as read-only resources with very limited user interaction capabilities. The only way for interacting with pathway data is to resort to specific stand-alone applications designed for visualization and editing purposes. The most accepted pathway editing tools are Cytoscape [26], GenMAPP [27] and PathVisio [28]. Cytoscape is a free open source platform providing biological network analysis features and two-dimensional network visualization. Its open source and plugin-based architecture allows worldwide programmers to develop their own plugins, which largely contribute to extend the capabilities of the basic environment, resulting in a very versatile network analysis application. Cytoscape supports multiple network formats, data integration, literature mining, clustering, functional enrichment, network comparison and programmatic access [29].

At the moment only a few Cytoscape plugins or standalone applications that provide functionalities to import, merge and visualize complex regulatory networks and to enhance them with transcriptional, post-transcriptional and translational information from external data sources do exist. However they still suffer from several limitations that prevent their widespread application.

In this paper we introduce the Regulatory Network Enhancer (ReNE) plugin, a new Cytoscape 3.x plugin, which enables integration, merging, enhancement, visualization, and exporting of pathways from multiple repositories. It enables to import pathways from the KEGG and Reactome online repositories or from any standard OWL/XML data format that the Cytoscape import procedure accepts. ReNE also enables to enhance the imported networks with transcriptional, post-transcriptional and translational information. It therefore represents a very interesting tool to enable an easy integration of multiple biological information into a single network that can be then analyzed and explored using the large set of tools provided by the Cytoscape ecosystem.

## Materials and Methods

**Figure 1:**
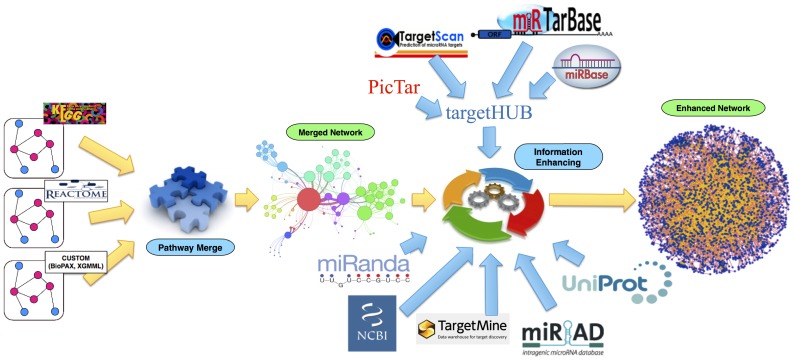
The ReNE data flow. KEGG, Reactome or custom pathways are retrieved and merged obtaining a merged network. The merged network is enhanced with information retrieved from multiple public repositories (i.e., miRIAD, TargetMine, miRanda, NCBI, UniProt, TargetHUB, PicTar, miRTarBase, TargetScan, miRBase). After the integration of all the new information, the resulting Enhanced Network is eventually provided.

Fig. 1 provides a very high-level picture of the ReNE workflow and main data sources. Overall, the plugin implements two main computational features identified as (i) *Pathway Merge* and (ii) *Information Enhancing*. The Pathway Merge feature enables scientists to automatically import networks available on well established online repositories as well as custom networks described using standard OWL/XML data formats that the Cytoscape import procedure accept (e.g., BioPAX, XGMML), and to merge them into a single *Merged Network*. The informative content of the obtained network can be then enhanced through the Information Enhancing engine that retrieves transcriptional, post-transcriptional, and translational data from several on-line repositories and integrate them in the network. The result is an enhanced network with increased informative content and ready for further automatic and visual analysis.

Additional details regarding the ReNE computational capabilities are provided in the next subsections.

### Pathway Merge

ReNE resorts to the internal Cytoscape import procedure to import any network described using a standard OWL/XML data format (e.g., BioPAX [24] and XGMML [30]) that Cytoscape is able to parse. This enables to process custom networks defined by the user as well as public networks available in a OWL/XML format (e.g., SignaLink2 networks [19]). Moreover, to improve the usability of the plugin, a dedicated user interface to browse and import pathways hosted on Reactome [17] and KEGG [16], resorting to the web services provided by these largely used repositories is also implemented.

**Figure 2:**
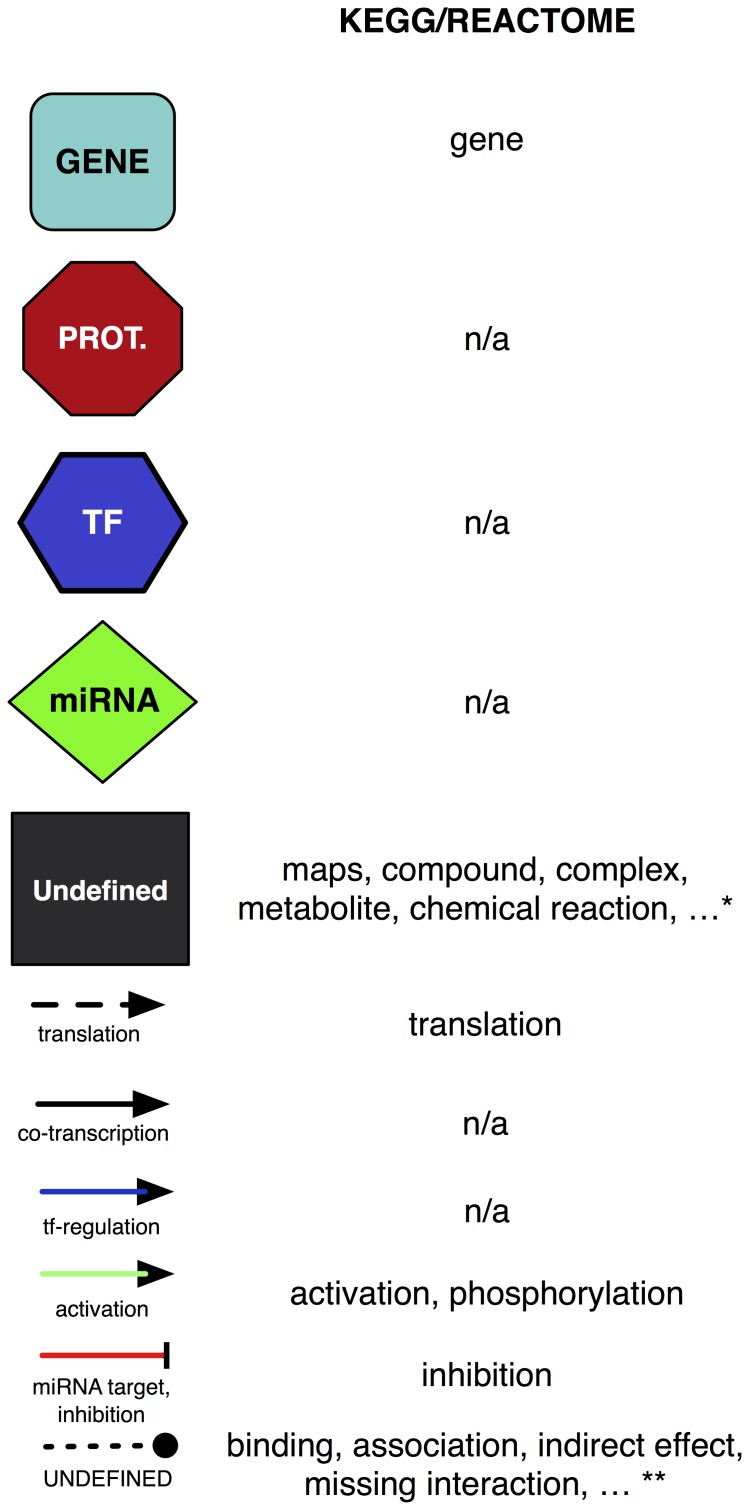
ReNE symbol list. For each symbol, the corresponding mapping rule for KEGG and Reactome is also provided. Some nodes and edges, mapped as "n/a", are not present in publicly available pathways and are generated by ReNE. * and ** correspond to node/edge definitions that are not informative for the plugin purpose. Such entities are mapped maintaining the original definition when possible, otherwise they are labeled as "Undefined".

One of the main challenges faced when importing and merging networks from heterogeneous databases is to integrate a large set of network symbols that are specific of the selected network source. ReNE exploits the symbol list reported in Fig. 2 that is general enough to represent most of the mechanisms usually modeled by available biological networks. Custom models defined by repositories like KEGG and Reactome are mapped to this generic symbol list in order to obtain a uniform network representation. This is particularly important when multiple pathways from different repositories are merged.

The ReNE import and merging process is lossless. Even when nodes and arcs are mapped to the ReNE symbol list, all information items available in the original network are stored as additional attributes to the nodes/arcs and are available to the user for later analysis.

### Information Enhancing

Networks obtained from the Pathway Merge processing can be enhanced with additional information gathered from a large set of external biological databases. This section overviews the data sources used by ReNE for network enhancing. Let us consider the simple network reported in Fig. 3.1 depicted using the ReNE symbols. The enhancing engine basically enables three enhancing options:

**Figure 3:**
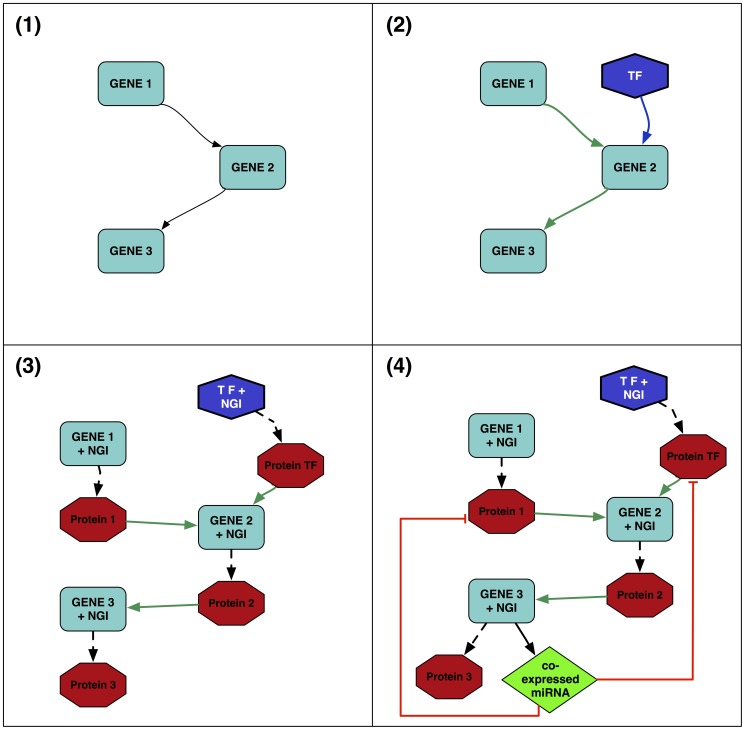
The image shows the set of enhancing steps automatically performed by ReNE. (1) The original regulatory network. (2) Transcriptional regulation enhancement: Transcription Factors coding genes are added to the network. (3) Translation regulation enhancement: genes are normalized in terms of symbols and accessions retrieving information from NCBI, while the network is enhanced with the protein coded by the genes retrieving information from Uniprot. (4) Post-transcriptional regulation enhancement: each gene is analyzed for discovering possible co-expressed intragenic miRNAs. For each miRNA, the list of its target genes is intersected with the network entities, and for each match the network is enhanced with a regulatory edge.

1. transcription factors enhancing (Fig. 3.2)
2. translational enhancing, i.e., normalization of gene/protein information (Fig. 3.3), and,
3. post-transcriptional enhancing (Fig. 3.4).

It is worth to highlight here that the execution of option (3) requires the information introduced by the execution of option (2). The execution of option (1) to include additional transcription factors is instead optional. This considers the case in which the initial network already contains transcription factors that do not require to be further enhanced.

#### Transcription factors enhancing

Transcription Factors (TFs) related to gene entities are retrieved from TargetMine [31]. Data from TargetMine are browsed through a RESTful interface, which allows users to directly search for TFs given a target gene. The RESTful interface provides results in a tab-separated format. Results are then parsed and integrated in the network by creating new TF nodes with outgoing up-regulatory edges directed to their target genes (Fig. 3.2).

#### Translational enhancing

Since different databases use different conventions to identify specific genes and proteins we resort to NCBI [32] and Uniprot [33] in order to obtain unique identifiers for these entities. Almost all pathway repositories include a standard Gene Symbol among the information associated to a gene. For each gene of the network, we use the Gene Symbol to query NCBI and to retrieve its standard NCBI GeneID together with additional genetic information items like the genomic coordinates that are stored as additional attributes of the network nodes. The GeneID of each gene is then used to univocally query Uniprot to obtain information related to the protein (if any) that the gene encodes. Data obtained from Uniprot include the protein's UniprotID used as accession number together with additional attributes (like protein isoforms) that are downloaded and integrated in the data model. For each gene encoding a protein, a new protein node is created in the network and connected to the related gene. This allows ReNE users to better inspect the intermediation of proteins in a regulatory network (Fig. 3.3), and to prepare the network for the introduction post-transcriptional information.

#### Post-transcriptional enhancing

Post transcriptional regulation enhancing is performed in two steps:

1. identification of intragenic miRNAs co-expressed with host genes available in the selected network, and
2. identification of the target genes of the miRNAs found in step one.

To identify the intragenic miRNAs hosted by a selected host gene, ReNE resorts to the miRIAD Intragenic Microrna database [34]. Intragenic miRNAs selected in this way are known to be co-expressed with their host genes [35]. The miRIAD website is a web search tool developed with the primary purpose of integrating relevant information concerning intragenic miRNAs and their host genes. The miRIAD database references annotated genes from human genome (hg19) and miRNAs annotated from miRBase (version 19). The mapping of intragenic miRNAs was performed according to [36].

At each run, ReNE sends a request to retrieve, deflate and parse miRIAD data available for download in a zipped text file, thus obtaining the whole list of miRNAs coupled with their host genes. For each miRNA hosted by one of the network genes, ReNE creates a new node and adds it to the network in order to better visualize the dependency between the host gene and its intragenic miRNA.

To conclude the post-transcriptional regulatory enhancement, for each identified miRNA the tool searches for the list of its target genes. To allow users to search miRNA target genes with the highest level of freedom we integrated in ReNE the primitives offered by TargetHUB. This web-service, based on a CouchDB database, provides a programmer friendly interface to access multiple repositories of miRNA target genes with a uniform set of APIs [13]. TargetHUB RESTful interface allows users to interrogate information from four different databases: miRTarBase [9], TargetScan [10], PicTar [11], and miRanda [12]. User defined search filters can be applied to limit the search to specific databases or selecting as valid target genes only those returned by at least a specified number of databases. Using TargetHUB, a list of target genes is retrieved for each miRNA discovered in the first step of the post-transcriptional enhancement. Each list is then intersected with all gene entities already present in the network and for each match a new edge is created, linking the miRNA entity to its target protein with a silencing edge (Fig. 3.4). The edge represents the post-transcriptional effect of the miRNA, which does not silence the transcription of its target gene, but instead blocks the transcript (mRNA) translation into a protein.

### ReNe user interface

**Figure 4:**
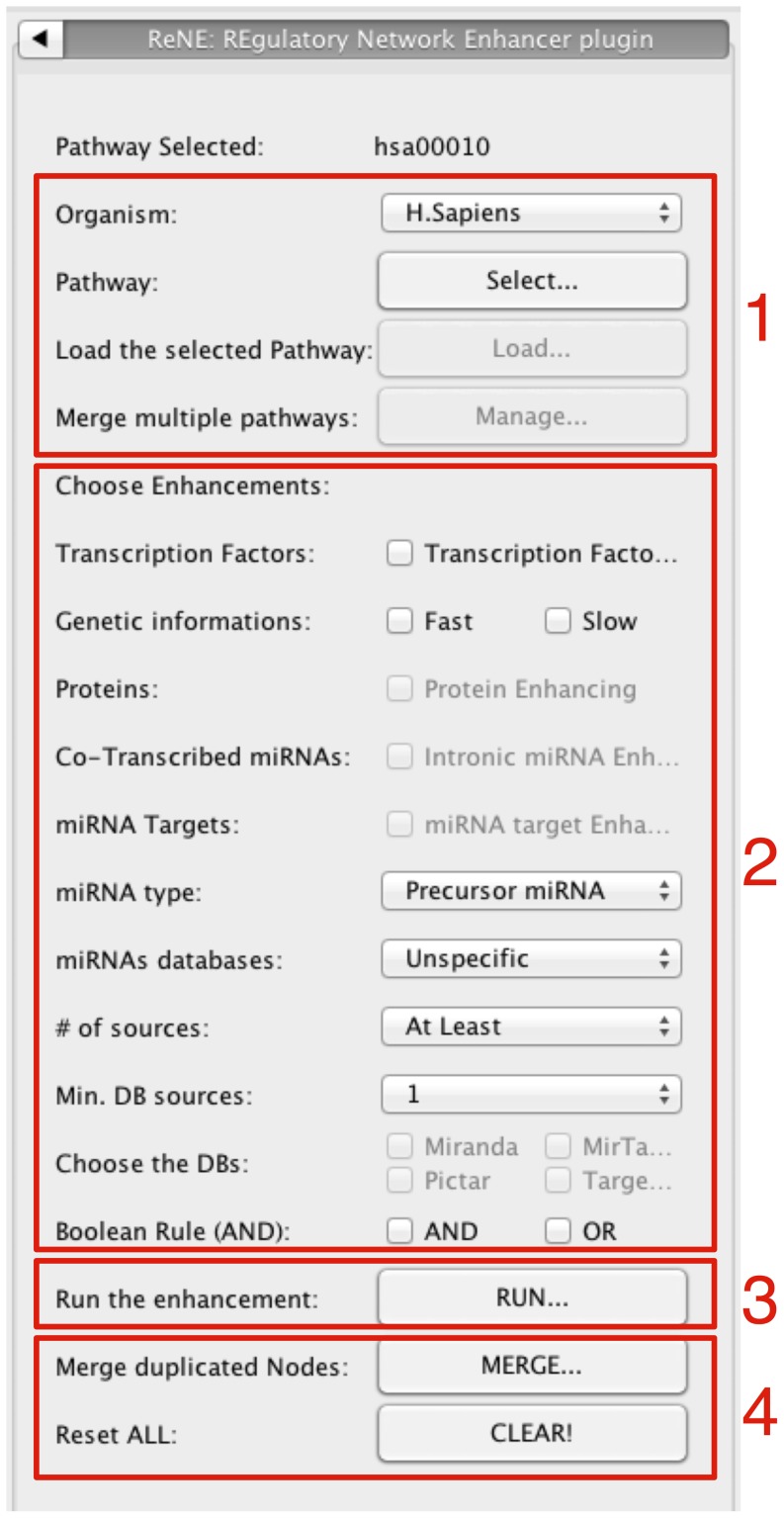
The image shows the ReNE control panel. Buttons are grouped according to their purpose: (1) Source pathway(s) management; (2) Available enhancements; (3) Run button for executing the selected enhancement; (4) Utility buttons for merging replicated nodes and reset the environment.

The entire pathway enhancement process is supported by a simple graphic user interface (GUI) that guides the user through all the necessary steps. To limit errors functionalities are enabled only when the operations they depend from have been executed. For example, before looking for miRNA target genes it is necessary to complete the network translational enhancement in order to have all potential miRNA targets already instantiated in the network. The user panel depicted in Fig. 4 shows the 4 groups of buttons available to control the plugin.

#### Pathway data upload, merging, and management

The buttons in group 1 allow the user to select the organism and the pathway(s) of interest. Using the Species selector the user can select among *H.Sapiens* and *R.Norvegicus* species that are currently supported by ReNE. The *Pathway select* button opens a popup window that enables to select pathways available in Reactome and KEGG either browsing a pathway list, or directly providing a pathway accession number. Custom pathways described using a standard OWL/XML data format can be also imported using this button. At this point the plugin imports the selected pathway and, by clicking the *Load* button, the corresponding Cytoscape network is created and visualized. If needed, further pathways can be added to the current network by using the *Manage* button. The popup window allows the user to add (or remove) pathways from the previous selections. It also allows the user to decide whether to merge or not the set of uploaded pathways. In case of merging, all genes which share the same GeneSymbol are merged in a single node and the network edges are remapped accordingly.

#### Pathway enhancing

When the initial network is properly loaded, the set of checkboxes in group 2 allows the user to choose the type of desired enhancement. Every time the user chooses a single enhancement, the *Run* button (group 3) is used to apply the task to the network. Group 4 buttons are basically utilities. The *Clear* button completely resets the current network, while the *Merge* button removes duplicated genes, TFs, proteins and miRNAs. No button to delete duplicate edges is provided since Cytoscape already offers a straightforward way for dealing with this problem. If needed the user can resort to the *Remove Duplicated Edges* option in the Cytoscape *Edit* menu.

#### Data download and export

ReNE, resorting to the editing and export capabilities of Cytoscape, allows the user to export the whole enhanced network, or part of it, to various graphical and machine-readable standard file formats (e.g., SBML, BioPAX, XGMML, Cys, SIF, tab-delimited and CSV). Moreover, ReNE supports the export of the network in the BNToolkit format. BNToolkit [37], [38] is a network simulation tool that enables to perform simulation and dynamic analysis of complex gene regulatory networks, enabling to inspect the underlying dynamics of the analyzed network. Further proprietary exporting file formats may be implemented on demand.

## Results

This section discusses the results obtained by the application of ReNE to the analysis of a selected pathway in order to highlight its the capability when applied to a realistic use case. The considered pathway is related to the Parkinson disease and comes from the KEGG pathway repository (KEGG ID: hsa05012, URL: http://www.genome.jp/kegg-bin/show_pathway?hsa05012). The original network downloaded from the KEGG repository in KGML format and imported in ReNE is available in S1 File, and the ReNE enhanced network is available in S2 File. It is worth to mention here that the goal of this section is not to present new results on the Parkinson research. It only aims at showing how ReNE could be a useful tool to help biologists finding explanations or formulating new hypothesis to explain biological data. In particular the selected example shows how, from the analysis of the Parkinson's disease network, even without any previous knowledge, it is possible to highlight a possible correlation between Parkinson's disease, mitochondrial dysfunctions and diabetes as formulated in [39]. In order to show that ReNE only introduces regulatory interactions from publicly available databases, preserving the informative content of the original network, the S3 File provides a manual validation of the enhancement process performed on the considered Parkinson's network.

Parkinson's disease (PD) is the second most common degenerative disorder of the central nervous system. PD is related to the death of dopamine-generating cells in the *substantia nigra*, a region of the midbrain, whose cause is still unknown. PD presents an early stage, in which symptoms are movement-related, and an advanced one, in which patients also experience impaired cognitive functions.

**Figure 5:**
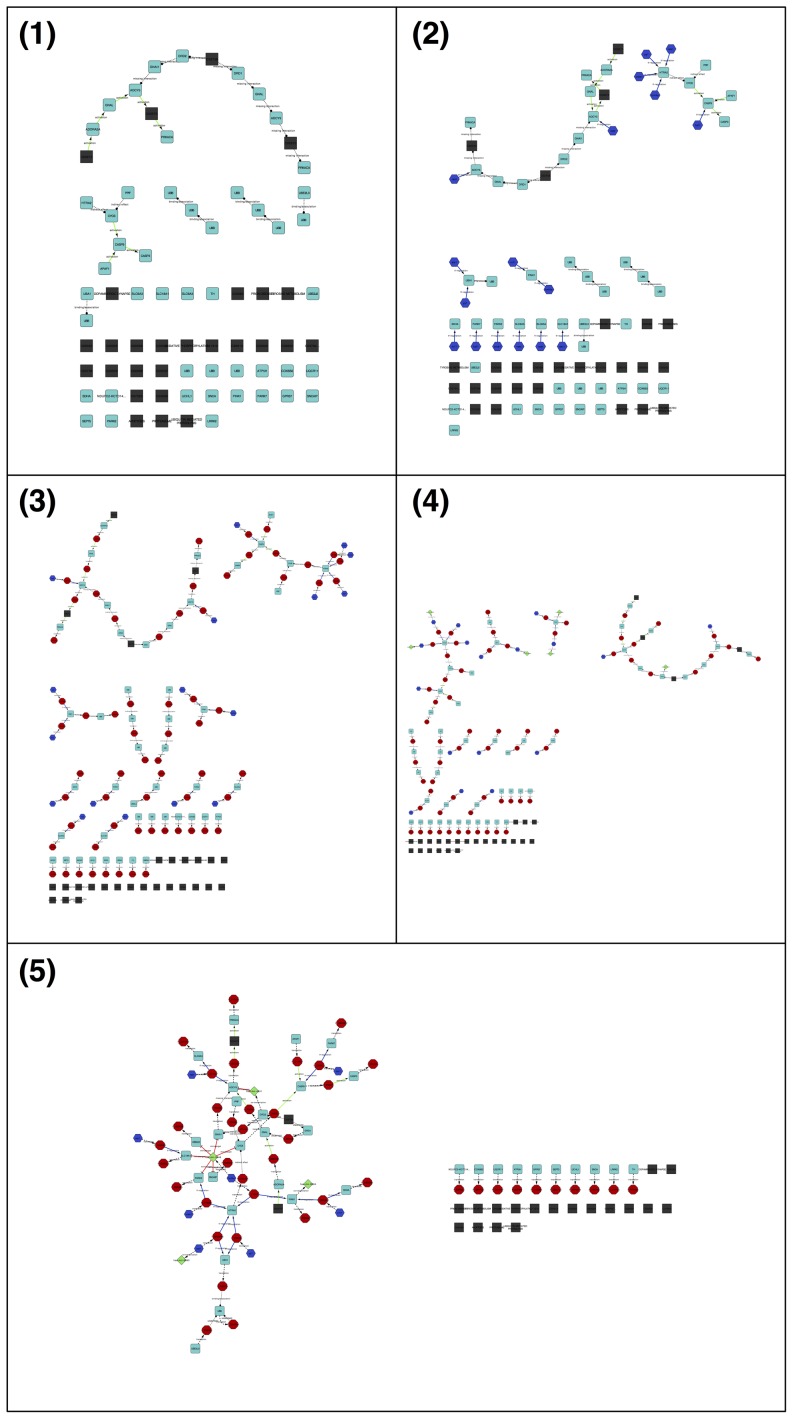
The image shows the network transformation after each enhancing step. (1) Original KEGG pathway as it appears after parsing of the KEGG Markup Language (KGMML) file. Cyan nodes are genes and black nodes are not-gene entities (i.e., maps, molecular compounds, etc.) (2) Transcription factors enhancing. TFs are formatted as blue hexagonal nodes. (3) Proteins, marked as red circles, are added to the network. (4) miRNAs co-expressed along with their host genes are inserted. miRNAs, marked as green diamonds, have incoming edges originated from their host gene. (5) The enhancing ends with the insertion of edges connecting the miRNAs found in previous step with their target genes.

Fig. 5 shows how the PD′s network layout changes after each enhancing step performed by ReNE. In the first step (see Fig. 5.1), the plugin downloads and parses the KGMML file describing the pathway from the KEGG repository. The reader may note the presence of several isolated nodes compared to the pathway picture available on the KEGG website. This is due to lack of information in the KGMML file and not related to the ReNE import procedure. Fig. 5.2 shows the network transformation after the introduction of Transcription Factors; Fig. 5.3 shows the insertion of proteins, while in Fig. 5.4 intragenic miRNAs co-expressed together with their host genes are inserted. Both genes (cyan squares) and TFs (blue octagons) translate into their corresponding proteins (red octagons), and both may co-express an intragenic miRNA (green diamonds). In the last step (see Fig. 5.5) every miRNA found in the previous step is then connected to its target proteins (if present in the network).

**Figure 6:**
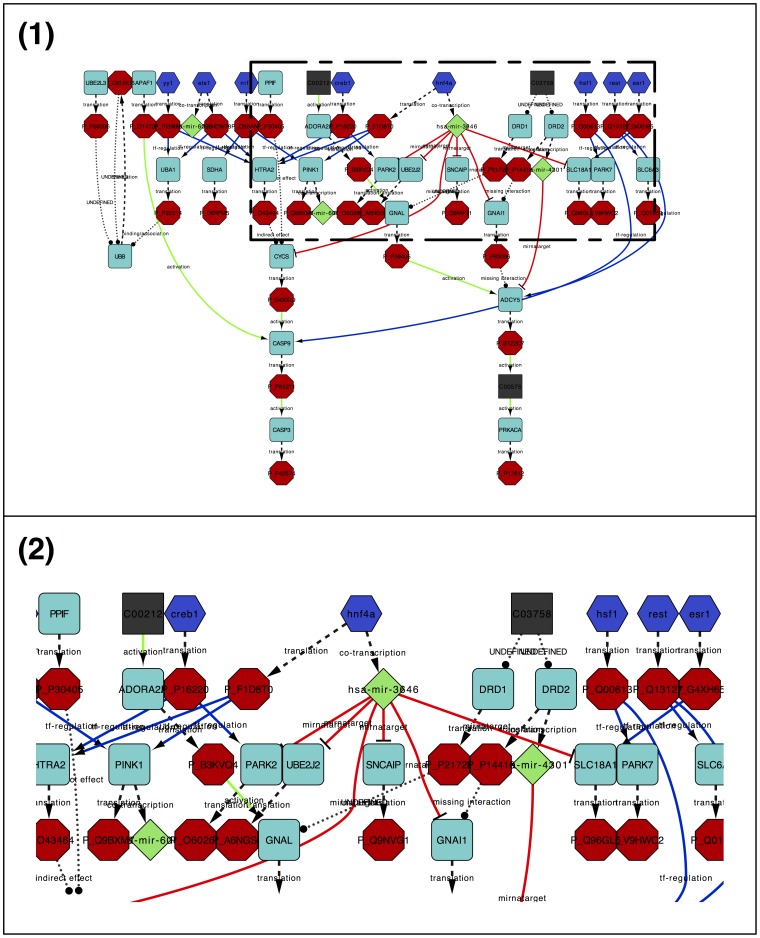
The image shows the Parkinson Disease network obtained from KEGG (ID: hsa05012), after its complete enhancing and layouting. (1) The entire enhanced network. (2) Zoomed section highlighting the role of *HNF4A* as mediator of *miR-3646* acting as hub for the inner regulation of the pathway.

After the last enhancing step, the ReNE utility button can be used to remove duplicated nodes and/or edges, if any, and the layout is changed into the Hierarchic layout, available in Cytoscape under the yFiles layout submenu (see Fig. 6.a). The Hierarchic layout allows to better appreciate the causality in the signaling cascade. Nodes are layered according to their relations. Therefore, we are likely to have source nodes at the top layers, and effectors or output of the signaling cascade at the bottom layers. Thus, changes in top layered nodes are more likely to affect the entire signaling cascade, resulting in ectopic behaviors of the outputs.

Fig. 6.b highlights how an enhanced network may give clues of the functional role of unexpected entities. For instance, *miR-3646* clearly emerges among other entities as a possible hub in the enhanced pathway. Given its high out-degree (i.e., the number of outgoing edges), *miR-3646* represents a very interesting node to be analyzed as possibly involved in PD. No information related to this miRNA is available in literature or in public repositories. Nevertheless, the miRNA host gene is the gene *HNF4A*, which encodes a TF deputed to control the expression of pancreatic, kidney, and liver islet-specific genes. *HNF4A* is also known to be highly involved in Maturity Onset Diabetes of the Young (MODY) [40]. Although apparently MODY and diabetes seem unrelated to PD, recently some researchers proved a relation between diabetes and PD [41], [42] and, even more interestingly, they discovered that *HNF4A* is responsible for the disregulation of several PD biomarkers [43]. Curiously, in [44], the authors did not find any noticeable expression of *HNF4A* in cerebral cortex and, in [45], authors also point to *HNF4A* as a major hypoxia-responsive transcription factor in hindbrain, but predominantly down-regulated in mid/forebrain. Given those premises, the enhanced network suggests the mediation of *miR-3646* as effector of the induced regulation in brain areas in which *HNF4A* is not generally expressed. miRNAs have been in fact already demonstrated to be able to migrate across tissues thanks to the exogenous vesiculation.

A second interesting entity introduced after the enhancement is *NRF1* that acts as transcription factor for *PINK1*. *NRF1* is considered among the earliest signs of insulin resistance and is involved in mitochondrial biogenesis and mitochondria-induced apoptosis, which is a prominent feature of neurodegenerative diseases including PD. In fact, in [39], authors observed that insulin resistance in PD brain would be caused by mitochondrial dysfunctions, which are, in turn, induced by *NRF1*. It causes *PINK1* accumulation that eventually induces *Parkin* to initiate the autophagic degradation of the damaged mitochondria.

Also *CREB1*, another entity added in the enhanced network, is an interesting PD-related candidate molecule and appears connected to both *HTRA2*, a gene coding for an enzyme involved in mitochondria stress-control, and *PARK2*, a component of the ubiquitin-proteasome system that mediates the targeting of proteins for degradation. While *CREB1* is well known for regulating neuronal differentiation, survival, and plasticity in the brain and peripheral nervous system, recently, in [46], researchers also suggested that *CREB1* may influence high-order cognitive functions by participating in brain adaptation to over-nutrition, which is eventually correlated to accelerated brain aging and diabetes.

## Discussion

ReNE is a new Cytoscape application that enables a very quick enhancement of biological regulatory networks with additional transcriptional, post-transcriptional and translational information. Two main design guides characterize ReNE. First it integrates information from a very extensive set of public repositories, thus enabling to embed a significant informative content within a single network. Second, it provides a very efficient and intuitive user interface that automates complex tasks and makes the application a potential software instrument for biologists with limited skills in computer programming and network analysis.

As already mentioned in the introduction of this paper at the moment a few Cytoscape plugins that provide functionalities somehow similar to those provided by ReNE do exist. Among them, the ones that are more related to ReNE are CluePedia [47], ConReg [48], miRScape and CyTargetLinker [49].

The CluePedia Cytoscape plugin is a search tool for new markers potentially associated to pathways. CluePedia elaborates experimental data to identify linked genes, proteins and miRNAs that are then integrated into a network with ClueGO [50] terms/pathways. The ConReg Cytoscape plug-in is not actually a network enhancer plugin. It enables to access the ConReg network repository, which includes conserved regulatory networks for major eukaryotic model organisms like *Human*, *Mouse*, *Fly*, and *Yeast* that are already enhanced with information about transcription factors obtained from several repositories. miRScape is a Cytoscape plugin allowing mining on biological networks annotated with miRNAs. It makes use of the knowledge base miRo [51], which introduces a new layer of associations between genes and phenotypes based on miRNA annotations. Given a network, previously loaded into Cytoscape, miRScape identifies relationships among genes, processes, functions and diseases at the miRNA level and annotates them as attributes of each network node.

Differently from ReNE, these three plugins focus on very specific types of regulatory interactions. CluePedia focuses on interactions that are extracted from experimental data provided by the user rather then integrating the huge amount of knowledge available in public repositories. ConReg focuses only on interactions between genes and transcription factors while miRScape only focuses on interactions among miRNAs and their target genes. Moreover miRScape has not been maintained since version 2.6 of Cytoscape released in 2010. Despite they provide interesting functionalities, they somehow lack the generality of ReNE that enables to considers several types of interactions within the same plugin.

CyTargetLinker is one of the most recent Cytoscape plugins that offers functionalities related to ReNE. CyTargetLinker is able to process a network already imported into Cytoscape. One of the main similarities with ReNE is that it enables to add regulatory interactions (e.g., miRNA target genes) to selected categories of nodes of the network. The new interactions are retrieved from a set of Regulatory Interaction Networks (RegINs) files that can be downloaded from the developers website or created in a custom way by the user. The use of RegINs files as a knowledge base represents a main difference compared to the enhancement process proposed by ReNE. The main advantage of the RegINs files is that they can be updated by the user, thus enabling to describe user defined interactions that must be included in the enhanced network. However, when it comes to the integration of data coming from public repositories that are continuously updated, RegINs files published by the CyTargetLinker developers may become quickly outdated and unless a constant effort to publish new releases of these files is provided, networks enriched with CyTargetLinker may lack some of the latest finding available in public repositories. Moreover ReNE offers a set of additional functionalities compared to CyTargetLinker. Multiple networks imported from different data sources can be easily merged to form a single integrated network. Moreover, automatic connection to public pathway repositories such as KEGG and Reactome provide a very simple user interface to quickly import public networks to analyze. Finally, during the network enhancement process ReNE do not simply add new interactions to the network. It carefully restructure the network in order to clearly model transcriptional, post-transcriptional and translational activities thus providing a better description of the biological activities described by the network.

A few stand alone applications providing functionalities related to ReNE are also available. Among them SignaLink2 [19] and TranscriptomeBrowser [52] are interesting related applications. The idea behind SignaLink2 is very interesting and goes in a direction very close to ReNE. However, even if SignaLink2 may feature a large pool of databases to retrieve information, the analysis is limited to 7 pathways (namely RTK, Hedgehog, JAK/STAT, NHR, Notch, 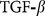, WNT/Wingless). There is no way for users to mine other pathway sources like Reactome or KEGG, neither to upload custom pathways to take advantage of SignaLink2 enhancing capabilities, thus limiting the use of this application to restricted cases. TranscriptomeBrowser is instead a large database of transcriptional signatures extracted from Gene Expression Omnibus which can be queried resorting to the TranscriptomeBrowser client and its plugin. The TranscriptomeBrowser client enables to query the TranscriptomeBrowser knowledge in order to infer regulatory interactions given a set of candidate genes. Through the InteractomeBrowser plugin the inferred regulatory interactions can be then displayed in a network format. Differently from ReNE, the tool does not enable to start from an already defined network and to enhance it with external data sources. Nevertheless networks created within TranscriptomeBrowser and exported in Cytoscape XML format can be imported and further processed using ReNE functionalities.

To the best of our knowledge, no other tools able to perform all the network enhancements provided by ReNE are available in the literature.

ReNE is an open source project available under a Creative Commons License (http://creativecommons.org/licenses/by-nc-sa/4.0/). It is freely available on the Cytoscape App Store (http://apps.cytoscape.org/apps/rene). The community can contribute to ReNE in different ways.

Programmers may directly help extending and improving the plugin functionalities. The full ReNE source code is freely available for download through a svn repository accessible at http://www.sysbio.polito.it/tools_svn/BioInformatics/Rene/releases/. The latest stable release of ReNE is v.1.5. New releases will be continuously published and released using the same URL. End users may also help by submitting suggestions, requests and notifications of bugs through the ReNE page at http://www.sysbio.polito.it/index.php/tools-and-downloads/item/220-rene. Support for the implementation of user-requested features such as non-standard output formats, import from specific databases or third party layout algorithms is also available on request from the ReNE team.

## Conclusions

ReNE is an ongoing project. We are continuously extending the set of functionalities and the set of databases available from the plugin. Since online repositories are continuously updated and new repositories are created very frequently we will work yo maintain the set of functionalities as update as possible, and to support more data sources, thus allowing users to have a larger spectrum of enhancing possibilities. The ReNE developers are also interested in projects supporting standardization of biological network descriptions such as the Biological Expression Language (BEL) project (http://www.openbel.org/)) to enable an easy interaction of ReNE with other tools supporting the same standard languages.

Already planned enhancements are the integration of additional pathway repositories, support network enhancing for additional species beside *Homo Sapiens* and *Rattus Norvegicus*, a better integration of Boolean Network analysis for pathway simulation, and data management capabilities.

## Supporting Information

S1 File **Cytoscape session file (cys) containing the Parkinson disease network dowloaded from KEGG in KGML format and imported in ReNE.**
(CYS)

S2 File **Cytoscape session file (cys) containing the Parkinson disease network enhanced by ReNE.**
(CYS)

S3 File **Excel file containing a manual validation of the enhancement process performed by ReNE on the Parkinson disease network.**
(XLSX)
